# Expression of MicroRNAs in the NCI-60 Cancer Cell-Lines

**DOI:** 10.1371/journal.pone.0049918

**Published:** 2012-11-28

**Authors:** Santosh K. Patnaik, Jesper Dahlgaard, Wiktor Mazin, Eric Kannisto, Thomas Jensen, Steen Knudsen, Sai Yendamuri

**Affiliations:** 1 Department of Thoracic Surgery, Roswell Park Cancer Institute, Buffalo, New York, United States of America; 2 Medical Prognosis Institute, Hørsholm, Denmark; 3 Department of Surgery, State University of New York, Buffalo, New York, United States of America; Virginia Commonwealth University, United States of America

## Abstract

The NCI-60 panel of 60 human cancer cell-lines of nine different tissues of origin has been extensively characterized in biological, molecular and pharmacological studies. Analyses of data from such studies have provided valuable information for understanding cellular processes and developing strategies for the diagnosis and treatment of cancer. Here, Affymetrix® GeneChip™ miRNA version 1 oligonucleotide microarrays were used to quantify 847 microRNAs to generate an expression dataset of 495 (58.4%) microRNAs that were identified as expressed in at least one cell-line of the NCI-60 panel. Accuracy of the microRNA measurements was partly confirmed by reverse transcription and polymerase chain reaction assays. Similar to that seen among the four existing NCI-60 microRNA datasets, the concordance of the new expression dataset with the other four was modest, with mean Pearson correlation coefficients of 0.37–0.54. In spite of this, comparable results with different datasets were noted in clustering of the cell-lines by their microRNA expression, differential expression of microRNAs by the lines’ tissue of origin, and correlation of specific microRNAs with the doubling-time of cells or their radiation sensitivity. Mutation status of the cell-lines for the *TP53, PTEN* and *BRAF* but not *CDKN2A* or *KRAS* cancer-related genes was found to be associated with changes in expression of specific microRNAs. The microRNA dataset generated here should be valuable to those working in the field of microRNAs as well as in integromic studies of the NCI-60 panel.

## Introduction

Systematic studies of sets of cell-lines have provided insights on biological processes and their mechanisms, and have proved useful for devising diagnostic and therapeutic approaches. For instance, phosphatase and tensin homologue (PTEN)-dependent and -independent tumor suppressor mechanisms have been identified using a panel of melanoma cell-lines [1], and genetic markers of sensitivity to the anti-cancer drug, trastuzumab (Herceptin™) have been delineated using a panel of breast cancer cell-lines [2]. The NCI-60 panel of 60 human tumor cell-lines of diverse histologies and nine different tissues of origin was developed by the National Cancer Institute (NCI) of USA in the late 1980s for the purpose of screening anti-cancer drugs [3]. Over the last two decades, the NCI-60 cells have been used to test more than 150,000 chemical compounds and natural product extracts for drug discovery [4]. Numerous studies have profiled the cells for phenotypic features, such as doubling time, drug efflux, and radiation sensitivity, and for characterization of their genomes and levels of RNA, proteins and metabolites [5], [6], [7]. Analyses of data integrated from multiple studies have also yielded significant insights of biological and clinical importance [8].

MicroRNAs, short non-coding RNAs, play significant roles in cellular processes by targeting protein-encoding mRNA transcripts to inhibit translation or cause their degradation. In humans, 2,042 species of mature microRNAs have been identified as per the latest version (19; August 2012) of the miRBase microRNA sequence repository [9]. Dysregulated expression of microRNAs occurs in many diseases, including cancer [10], and this observation has led to multiple studies demonstrating therapeutic and biomarker utility of these molecules [11], [12]. Evaluation of the microRNAs expressed in the NCI-60 panel of cell-lines has proved useful in identifying, among other things, biological roles of specific microRNAs [13], [14], the mechanistic bases and biomarker potential of microRNAs in drug sensitivity [15], [16], genetic signatures for diseases [17], [18], and the structural underpinnings of microRNA regulatory networks [19], [20].

The expression of microRNAs in the NCI-60 cells were first quantified in 2007 by Gaur, et al. [21] and Blower, et al. [22] in studies that respectively examined 241 and 321 species of the molecules. The current study was designed to expand the NCI-60 microRNA expression profiles to cover the many hundreds of microRNAs that have since been discovered. While this study has been in progress, two other groups have published data quantifying >700 microRNAs in the NCI-60 panel [23], [24]. Here, besides the generation and characterization of a new microRNA expression dataset, such multiple datasets are compared, and analyses that integrate information on cell proliferation, mRNA expression, oncogene mutations, or radiation sensitivity from studies of the cell-line panel are reported.

## Materials and Methods

### Isolation of RNA from Cells of the NCI-60 Panel

For each of the 60 cell-lines of the NCI-60 panel (table S1), one ml of frozen cells (1–2×10^6^) in cell-line-specific culture medium containing 10% dimethylsulfoxide was obtained from the Developmental Therapeutics Program (DTP) of the Division of Cancer Treatment and Diagnosis of the NCI. Immediately prior to RNA isolation, cells were thawed in a water-bath at 37 °C for five minutes and collected by centrifugation at 200 g for five minutes at room temperature. Isolation of total RNA from cells using the miRVana™ kit (Ambion®, Austin, TX) was done in randomized batches of 12–24 cell-lines over four days within 10 days of obtaining the cells. RNA concentration was measured by absorbance spectrophotometry on a NanoDrop™ 2000 instrument (Thermo Scientific®, Waltham, MA).

### Quantification of microRNAs by Reverse Transcription-PCR (RT-PCR)

Levels of mature microRNAs *miR-16*, *−21*, *−30a-5p*, *−106a* and *−200b* in 50 ng total RNA were measured using TaqMan™ microRNA reverse transcription kit and RT-PCR assays (Applied Biosystems®, Foster City, CA) as per protocols suggested by the manufacturer. Identification numbers of the microRNA assays were 391, 397, 417, 578, and 1800, respectively. RT and PCR reactions for all RNA samples were performed together for each microRNA assay. Cycle quantification (C_q_) values were calculated as the average of C_q_ values identified by SDS software (version 2.3; Applied Biosystems®) for triplicate 40-cycle PCR reactions that were performed in a 7900HT thermocycler (Applied Biosystems®).

### Microarray Hybridization

The GeneChip™ miRNA array (version 1; Affymetrix®, Santa Clara, CA) platform was used to quantify microRNAs in total RNA isolated from the NCI-60 cells. One µg RNA was polyadenylated and ligated with biotinylated 3DNA™ dendrimers using the FlashTag™ Biotin HSR RNA Labeling Kit (Genisphere®, Hatfield, PA). The labeled RNA, in a volume of 80 µl, was heated to 99°C for 5 minutes and then to 45°C for 5 minutes for loading on an array using material and methods provided with the Affymetrix® GeneChip™ Hybridization, Wash and Stain kit. RNA-array hybridization was performed with agitation at 60 rotations per minute for 16–18 hours at 48°C on an Affymetrix® 450 Fluidics Station, after which arrays were washed and a streptavidin-phycoerythrin conjugate was used for the detection of bound RNAs on the arrays with a GeneChip™ Systems 3000Dx2 (Affymetrix®) confocal laser-scanning microscope. Signal values were computed using the Affymetrix® GeneChip™ Command Console software. Data from all the arrays had low background noise, a similar distribution of signals for endogenous RNAs, and a similar and concentration-dependent signal distribution for specific RNAs that were spiked in the RNA samples before they were labeled. The raw signal data has been deposited with accession number E-MTAB-327 in the ArrayExpress database of the European Bioinformatics Institute [25].

The GeneChip™ miRNA version 1.0 array has 11 µm-sized 46,228 probe-spots for 7,815 DNA oligonucleotide probes including those against 922 non-microRNA human RNAs, 847 human microRNAs, and 5,856 microRNAs of 70 other species. Probes to detect microRNAs are spotted on the array in quadruplicate. The array also has 95 non-specific probes, each spotted up to 94 times, that are binned by GC content. Signals from these background probes are used to make ‘absent’ or ‘present’ detection calls. In total, 72 array hybridizations were performed in nine batches, each with eight arrays, over three days, with two, three and four batches used on the first, second and third day, respectively (table S1). Quadruplicate hybridizations were performed for four arbitrarily chosen cell-lines to assess technical replicability (table S1).

### Microarray Data Processing

Raw data files from the 72 array hybridizations were processed together using Affymetrix® miRNA QC Tool software (version 1.0.33) with the following steps in order as suggested by Genisphere®: background detection, background correction with the robust multichip average (RMA) method [26], quantile normalization, and median polish summarization [27]. Quality control and inter-replicate correlation analyses were used to identify the best of the four replicates for four of the RNA preparations (four cell-lines) that were assayed in quadruplicate. Data from the remaining three replicates were excluded from the final expression dataset. At this stage, the expression data matrix had signal values for 7,635 human as well as non-human RNAs. Values for 324 non-microRNA RNAs and 2,282 microRNAs with ‘absent’ detection calls for all 60 cell-lines were then removed from the data matrix. Expression of RNAs with signal values <4x the average inter-quartile range of signal values for all RNAs was considered as absent, and RNAs whose expression was absent in all cell-lines were then excluded from further analyses.

### Comparison with Existing NCI-60 MicroRNA Expression Datasets

Pre-processed microRNA expression datasets generated by Blower, et al. [22], Gaur, et al. [21], Liu, et al. [24], and Sokilde, et al. [23] were respectively obtained using NCI’s CellMiner™ web application [28], the DTP website, the authors, and Gene Expression Omnibus of the National Center for Biotechnology Information, USA, with accession number GSE26375. For the dataset of Gaur, et al., the row-names for microRNAs were standardized to the names used in version 15 of the miRBase database [9] as has been described previously [23]. Pearson correlation coefficients between untransformed microRNA measurement values in these datasets and in the one generated in this study were calculated using R.

### Correlation Analyses of Levels of microRNAs and their Target mRNAs

Raw gene expression data acquired using Affymetrix® GeneChip™ HG-U133A DNA oligonucleotide microarrays for the expression of mRNAs in 57 of the 60 NCI-60 cell-lines of this study could be obtained using the CellMiner™ web application. A list of 10,392 conserved mRNAs (unique gene symbols) predicted by the TargetScan 5.1 algorithm [29] to be targets of 153 conserved microRNA families was obtained online at http://www.targetscan.org. The mRNA expression data was pre-processed using the RMA method with the Affy Bioconductor package [30] in R, to obtain normalized and log_2_-transformed microarray signal values. A filter was then applied to retain values for only those probes for which the sample range of signal values was ≥1. Of the 495 microRNAs considered expressed in this study, 192, belonging to a total of 121 conserved microRNA families, were identified as targeting 6,465 of the 10,392 mRNAs. Gene symbols of the 6,465 mRNAs were converted to 10,612 Affymetrix® probe identifiers using the biomaRt Bioconductor package [31] in R. A total of 117,004 Pearson correlation coefficients between expression levels of the 192 microRNAs and their target mRNAs as detected by the 10,612 probes were then computed in R. Two-tailed t tests with Benjamini-Hochberg correction for a false discovery rate (FDR) of <5% were used to assess significance of the correlations. Correlations were also computed for 10 random permutations of class labels (identifying the cell-lines) of the mRNA expression dataset.

### Other

The limma (version 3.10.0) Bioconductor package [32] was used in R for analysis of differential expression using log_2_-transformed expression values. Statistical analyses and graphical plotting were done using Excel™ (version 12; Microsoft®, Redmond, WA), Prism™ (version 5.0c; GraphPad®, La Jolla, CA), and R (version 2.12.1). TM4 [33] MultiExperiment Viewer (version 4.6) was used for hierarchical clustering analysis with optimization for leaf-ordering. Unless stated otherwise, a P value below 0.05 in statistical tests was used to deem statistical significance. Details on correction for multiple testing are provided with the results of the analyses where such a correction was applied.

## Results

### MicroRNA Expression Profiling of NCI-60 Cells Using Affymetrix® Microarrays

GeneChip® miRNA 1.0 microarrays from Affymetrix® were used to examine the expression of 847 microRNAs in total RNA of 60 cell-lines of the NCI-60 cancer cell-line panel (table S1). This work was performed over three days in a total of nine batches of eight RNA samples each (table S1). RNA used for the expression profiling was obtained from frozen cells, similar to a previous study of microRNA expression in the NCI-60 panel [21]. The yield of RNA from 1–2×10^6^ cells of the cell-lines ranged from 11 to 60 ug (mean = 37; standard deviation [SD] = 11).

Technical replicability of the expression profiling method was confirmed by examination of hybridization data for four RNA preparations, one each from the KM12, PC-3, RPMI-8226 and OVCAR-8 cell-lines that were assayed in quadruplicate. The mean (and SD) of the 75th percentiles of log_2_-transformed microarray signal values for the quadruplicate sets for the 1,779 probes (847 for microRNAs) annotated as human-specific were 3.58 (0.07), 3.61 (0.12), 3.62 (0.04) and 3.50 (0.10), respectively. For the 95th percentiles, the values were 9.60 (0.10), 9.43 (0.09), 9.66 (0.11) and 9.60 (0.21), respectively. Mean (and SD) of the 12 self-self Pearson correlation coefficients for the four cell-lines were 0.98 (<0.01), 0.97 (0.01), 0.98 (<0.01) and 0.98 (0.01), respectively. For each cell-line, the quadruplicate hybridizations were performed on different days except that for OVCAR-8, one pair of the quadruplicates was assayed in the same batch (table S1). The Pearson correlation coefficient was 0.980 for this pair of intra-batch replicates.

At least one of the 60 cell-lines expressed 523 (63.4%) of the 847 microRNAs detectable by the microarray platform as indicated by the significantly higher signal values from the microRNA-specific probes compared to non-specific mismatch probes of similar GC content. Although the inter-replicate correlations described above appear excellent, a closer examination revealed that correlations were not good at low microarray signal values. This is shown in figure S1 for four inter-replicate comparisons. Since this indicated high noise levels at low signal values, a signal value-based filtering was done to identify RNAs that could be considered as being more precisely quantified. Accordingly, the expression of RNAs with signal values <4x the mean (18.3) of the inter-quartile ranges of signal values for all hybridizations (range = 13.3–26.3; SD = 2.7) was considered as absent. Twenty-eight microRNAs with expression absent in all 60 cell-lines as per this criteria were excluded from further analyses. Thus, 495 (58.4%) of the 847 microRNAs detectable with the microarray platform were considered expressed and the data for them was used for subsequent analyses.

### Correlation between Array- and RT-PCR-based microRNA Quantifications

**Figure 1:**
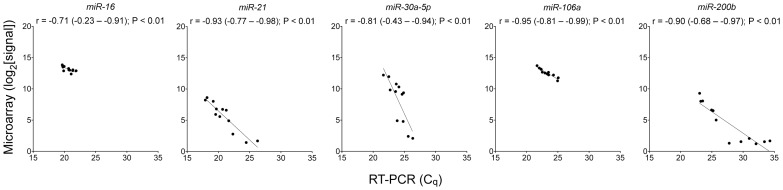
Confirmation of microarray-based microRNA quantifications by reverse transcription-PCR assays. Pearson correlation coefficients (*r*), P values, and best-fitting (least squares) lines are shown for RT-PCR C_q_ and log_2_-transformed microarray signal values for microRNAs *miR-16*, *−21*, *−30a-5p*, *−106a*, and *−200b* (n = 12).

To at least partially confirm the accuracy of the array-based microRNA expression measurements, RT-PCR was used to quantify levels of microRNAs *miR-16*, *−21*, *−30a-5p*, *−106a*, and *−200b* in 50 ng each of the RNA preparations for 12 cell-lines. The cell-lines were randomly selected and the microRNAs were arbitrarily picked from those for which at least half of the cell-lines had log_2_-transformed microarray signal value >5. As shown in figure 1, the two sets of measurements for all five microRNAs had significant correlations (P<0.01) in Pearson tests, with absolute correlation coefficient ≥0.70. The values of the slope of linear regression lines (least squares method) varied from *−*0.40 (*miR-16*) to *−*0.93 (*miR-21*) for four microRNAs, indicating better detectability of the microRNAs with the RT-PCR assay. For *miR-200b*, this is also notable with the five RNA samples with poor microarray signal values (2^1^.^2^–2^1^.^7^) but detectable RT-PCR C_q_ values in a good range (27.8–34.3). For *miR-30a-5p*, the slope of the regression line was *−*2.2, suggesting better detectability with the microarray platform.

### Comparison with Existing NCI-60 microRNA Expression Datasets

**Figure 2:**
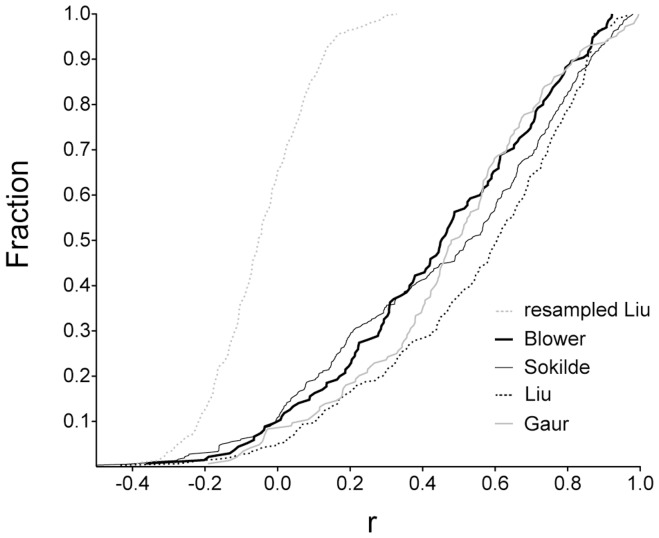
Correlations between different NCI-60 cell-line microRNA expression datasets. Cumulative frequency distributions are shown for Pearson correlation coefficients (*r*) with a bin-size of 0.025 for microRNAs quantified in this study and in the studies of Blower, et al. [22], Gaur, et al. [21], Liu, et al. [24], and Sokilde, et al [23]. The distribution of the coefficients with the expression measurements of Liu, et al. resampled is also shown.

Of the 495 microRNAs considered expressed in the set of 60 cell-lines in this study, 135 (27%), 144 (29%), 299 (60%) and 359 (73%) were also quantified in 59 of the 60 cell-lines in the studies of Blower, et al. [22], Gaur, et al. [21], Liu, et al. [24], and Sokilde, et al. [23], respectively. These four studies respectively used custom 40-mer DNA microarrays, TaqMan™ RT-PCR assays, Agilent® 60-mer DNA microarrays (version 2), and Exiqon® locked nucleic acid miRCURY™ microarrays (Dx, version 9.2) to assess the expression of 321, 241, 723 and 955 microRNAs, respectively. In Pearson correlation analyses of untransformed expression measurements of microRNAs common to this and the Blower, Gaur, Liu or Sokilde studies, the mean (and SD) of the Pearson coefficients were 0.44 (0.30), 0.47 (0.28), 0.54 (0.29) and 0.45 (0.34), respectively. The fractions of microRNAs with Pearson coefficient >0.75 were 0.18, 0.16, 0.29 and 0.24, respectively (figure 2). In all four comparisons, correlation coefficients were higher for microRNAs with high signal values than for those with low values (data not shown). Similar analyses were also performed to compare the datasets of Sokilde, et al. and Liu, et al. against others (figure S2). When correlating the Sokilde dataset with the Blower, Gaur and Liu datasets respectively, the mean (and SD) of the Pearson coefficients were 0.37 (0.32), 0.39 (0.33) and 0.44 (0.30), respectively. Values were 0.47 (0.31) and 0.42 (0.22) in comparisons of the Liu dataset with the Blower and Gaur datasets, respectively. Thus, judging by the correlations seen with existing datasets, the ‘correctness’ of the microRNA expression dataset generated in the current study is similar to the ones generated in previous studies. Because of variable sensitivity and specificity of the five quantification platforms of these studies for different microRNAs, a cross-platform comparison for cell-line correlations was not performed.

### MicroRNA Expression and Tissues of Origin

**Figure 3:**
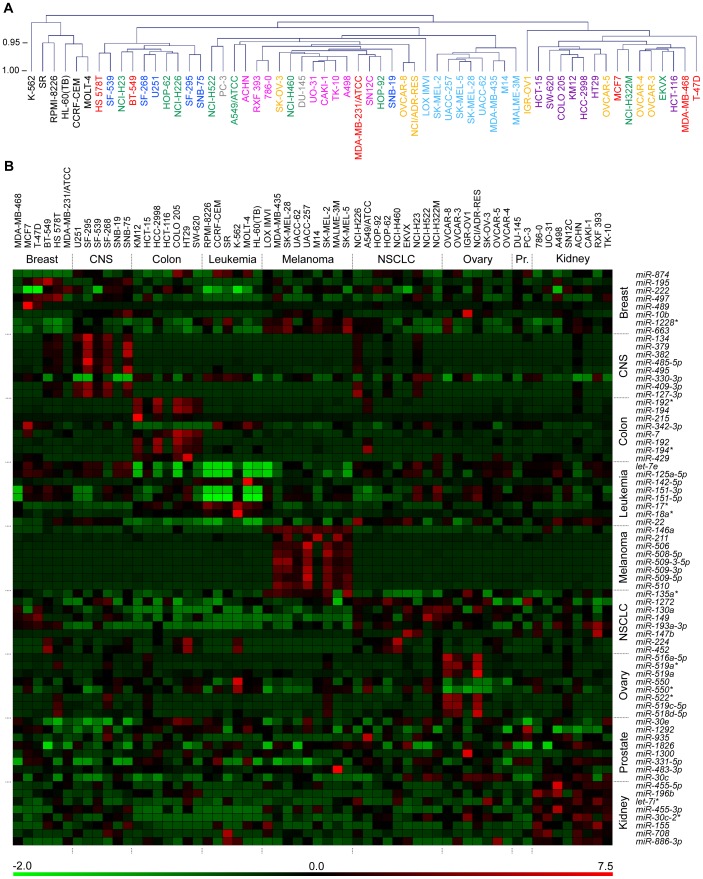
MicroRNA expression in the NCI-60 cell-lines by tissue of origin. *A*. Unsupervised clustering of 60 NCI-60 cell-lines by log_2_-transformed microarray signal values of the 495 expressed microRNAs is shown as a dendrogram. The different types of tissues of origin of the cell-lines are indicated by their color (*Pr.*, prostate). The tree is drawn from uncentered Pearson correlations, with average linkages used for joining clusters. The scale on the left represents node-heights. *B*. Heat-map, with its pseudo-color scale underneath, of Z scaled microarray signal values of the 60 cell-lines for the sets of eight microRNAs each with lowest P values in tests of differential expression in cell-lines of a specific tissue of origin compared to all the other cell-lines. Both cell-lines and microRNAs are grouped by tissue of origin. Z scaling was done along rows (by microRNA).

To assess if microRNA expression among the cell-lines was associated with the tissue of origin of the cell-lines, unsupervised hierarchical clustering based on average uncentered Pearson correlation was performed using log_2_-transformed microarray signal values for the 495 expressed microRNAs (figure 3A). The groups of leukemia and melanoma cell-lines completely segregated into their own independent clusters. All the seven but one (HCT-116) colon cancer cell-lines too segregated into one independent cluster. A similarly robust clustering of cell-lines for the remaining six tissues of origin was not seen. The OVCAR-8 ovarian cancer cell-line and the NCI/ADR-RES cell-line derived from it [34], as well as the M14 melanoma cell-line and the MDA-MB-435 cell-line derived from it [35], clustered as neighbors as expected. However, this was not seen for the third pair of homologous cell-lines in the NCI-60 panel, the U251 cell-line and its derivative, SNB-19 [36]. DNA fingerprinting suggests that, compared to the 94%–97% genetic similarity of the other two pairs, this pair of lines is only 81% similar.

To identify microRNAs differentially expressed in the cell-lines of a specific tissue-origin compared to all the other cell-lines, empirical Bayes moderated t-statistics and Benjamini-Hochberg correction for FDR <5% were used. The groups of cell-lines from CNS cancer (n = 6), colon cancer (n = 7), leukemia (n = 6), melanoma (n = 9), ovarian cancer (n = 7) and renal cancer (n = 8), respectively, showed differential expression of 83 (17%), 37 (7%), 137 (28%), 74 (15%), 14 (3%) and 3 (0.6%) of the 495 microRNAs present in the full dataset. No differentially expressed microRNAs could be identified in the groups of cell-lines from breast (n = 6), NSCLC (n = 9) or prostate (n = 2) cancer though the groups had 10 (2%), 24 (5%) and 4 (0.8%) microRNAs for which the P values were <0.05 without adjustment for false discovery. The heat-maps in figures 3B and S6 shows the expression of eight microRNAs for each of the nine groups of cell-lines with the lowest P values. Detailed statistics for the 72 total microRNAs are provided in table S2.

### MicroRNA Expression and Radiation Sensitivity

Amundson, et al. have examined the gamma radiation sensitivity of 59 of the 60 cell-lines of this study to quantify seven different radiation survival parameters [37]. The parameters include SF2, SF5 and SF8, the fraction of cell population that survives after exposure to 2, 5 and 8 Gy of radiation, respectively. To check if the NCI-60 cell-lines can be assorted into two radiation-sensitive and -resistant groups of similar size, as has been done for drug sensitivity (e.g., [6], [38], [39]), a non-parametric method was used to estimate Gaussian kernel densities of the radiation response data after log_2_ transformation and z-score normalization. A bimodal distribution with similarly-sized sensitive and resistant groups was not observed for any of the seven radiation survival parameters (figure S3). The radiation parameters were therefore considered as continuous variables and Pearson correlation analyses of log_2_-transformed parameter values with log_2_-transformed microRNA expression values were performed. Though significant correlations with absolute Pearson coefficient >0.5 were not seen for four of the radiation sensitivity parameters, they were present for 30 (6%), 27 (5%) and 33 (7%) of the 495 expressed microRNAs of this study for SF2, SF5 and SF8, respectively (figure S4). However, the correlations for all three parameters were largely driven by the six highly radiation-sensitive leukemia cell-lines of the NCI-60 panel. When the leukemia lines were excluded from the correlation analyses, expression of none of the 495 microRNAs correlated with absolute Pearson coefficient >0.5 for any of the three radiation sensitivity parameters. Similar results were seen when the microRNA expression datasets of Liu, et al. and Sokilde, et al. were analyzed (figure S4). As listed in table S3, 10, four and 14 microRNAs in the datasets of the current study, Liu, et al., and Sokilde, et al., respectively, had absolute Pearson coefficient >0.4 for at least one of SF2, SF5 and SF8. MicroRNAs *let-7i*, *miR-142–5p* and *miR-193b* were common to the results with the three datasets.

### Association of microRNAs with Oncogene Mutations

**Figure 4:**
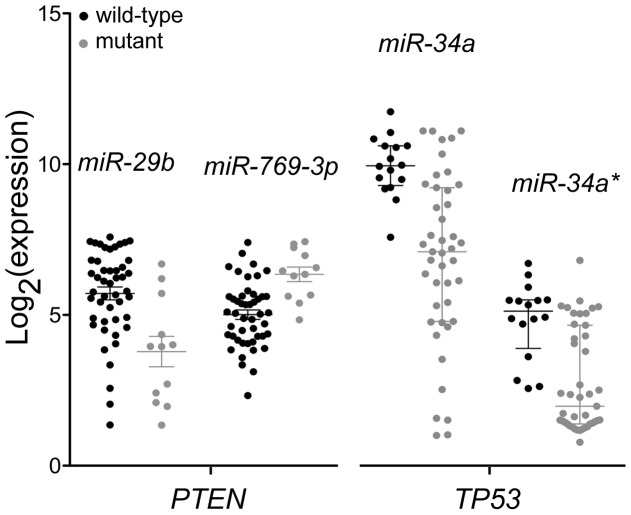
MicroRNAs and PTEN or TP53 mutations in NCI-60 cell-lines. Dot plots with medians and inter-quartile ranges are shown for the expression of microRNAs *miR-29b, −34a*, −34a* and *−769-3p* in 59 NCI-60 cell-lines grouped by mutation status for the *PTEN* or *TP53* genes.

The mutation status of 24 oncogenes is known for 59 of the 60 cell-lines of this study [40]. For some of the genes – *BRAF*, *CDKN2A* (*p16*), *KRAS*, *PTEN*, and *TP53*– oncogenic mutations exist in >10 of the cell-lines, which allows for a comparison of microRNA expression levels between the groups of mutant and wild-type cell-lines. Using empirical Bayes moderated t-statistics and Benjamini-Hochberg correction for FDR <5%, microRNAs *miR-29b* and *miR-769-3p* were found to be the only two microRNAs that were differentially expressed between the 47 mutant *PTEN* and 12 wild-type *PTEN* lines (P<0.01 for both microRNAs). In a similar analysis, microRNAs *miR-34a* and miR-34a* (*miR-34a-3p*) were found to be the only two microRNAs that were differentially expressed between the 43 mutant *TP53* and 16 wild-type *TP53* lines (P<0.01 for both microRNAs). The differential expression of the four *PTEN*- or *TP53*-associated microRNAs is depicted in figure 4. For the *BRAF* gene, for which 11 cell-lines carry mutations, 40 microRNAs were differentially expressed, with *miR-146a* and *miR-509-3p* overexpressed and *miR-149* and *mR-192b* underexpressed the most (table S5). This high number of differentially expressed microRNAs may be because eight of the 11 *BRAF* mutant cell-lines are melanoma cell-lines. No differentially expressed microRNAs were identified in such analyses for *CDKN2A* (33 mutant cell-lines) or *KRAS* (11 mutant cell-lines).

### Correlation between Levels of microRNAs and their Target mRNAs

Significant correlations between expression of microRNAs and mRNAs were detected for 3,483 (3%) of 117,004 possible pairs of microRNAs and their target mRNAs as predicted by the TargetScan algorithm. The 3,483 pairs consisted of 167 microRNAs and 1,232 target mRNAs with unique gene symbols. Among the 3,483 significant correlations, 1,997 (57%) were negative and 1,486 (43%) were positive (figure S5). In contrast, the mean and SD for the number of significant correlations seen with 10 random permutations of the mRNA expression dataset to switch cell-line identifiers were 21.3 and 6.6, respectively. With Benjamini-Hochberg correction for FDR below 5%, a mean value of 174 was expected. Furthermore, the values of 117,004 correlation coefficients obtained with the mRNA expression dataset were significantly different in Wilcoxon rank sum tests (P<2×10^−16^) from those obtained with any of the 10 permutated datasets.

## Discussion

The expression of 847 microRNAs in the NCI-60 panel of human tumor cell-lines was examined in this study using Affymetrix® GeneChip™ miRNA 1.0 DNA oligonucleotide microarrays to generate a dataset of 495 microRNAs identified as expressed in at least one of the 60 lines of the panel. Thus, the study expands the microRNA coverage of three of the four existing NCI-60 expression datasets. These sets were independently generated using different high throughput platforms – custom 40-mer DNA microarray, TaqMan™ RT-PCR, Agilent® 60-mer DNA microarray, or Exiqon® locked nucleic acid miRCURY™ microarray – to assess levels of 321, 241, 723 or 955 microRNAs, respectively [21], [22], [23], [24]. As depicted in figures 2 and S2, the correlation of microRNA expression measurements among the five datasets is modest, with mean Pearson coefficients varying from 0.37 to 0.54. These values, likely reflecting a known discordance among different microRNA quantification platforms [41], [42], highlight the usefulness of the dataset produced in this study in ascertaining results obtained using another dataset. Vast amount of information for a multitude of features exist and continue to be generated (e.g., [43], [44]) for the NCI-60 lines, and, as cited in the introduction section, integrated analysis of microRNA expression with other features of the cells has the potential to yield significant insights to generate and test hypotheses.

Unsupervised hierarchical clustering of the cell-lines by the microRNA expression patterns quantified in this study shows segregation by tissue of origin, and thus the relative homogeneity, of the lines derived from leukemia, melanoma or colon tumors (figure 3A). Similar clustering results are seen with the other NCI-60 microRNA datasets, even with that of Gaur, et al. which has expression measures for only 241 microRNAs [21], [22], [23], [24]. It is noteworthy in this regard that an assay of only 64 microRNAs has been found to quite accurately identify the tissue of origin of 42 different types of human tumors [45]. Analysis of the current study’s dataset for tissue-specific microRNA expression (figure 3B and table S2) shows that the highest degree of tissue-specific differences are seen for the leukemia, melanoma and colon cancer cell-lines, which interestingly cluster well by tissue of origin (figure 3A). Comparable tissue-specific microRNA expression patterns are identified using the five different NCI-60 microRNA expression datasets in spite of the modest inter-dataset correlations mentioned above. For instance, higher expression of microRNAs *miR-192* and *miR-194* in colon cancer cell-lines, and of *miR-708* and *miR-886-3p* in renal cancer cell-lines (table S2) was also observed by Sokilde, et al [23]. Similarly, higher expression of *miR-382* in the CNS cell-lines can be observed in the datasets of this (table S2) as well as the Blower and Gaur studies [21], [22]. The association of microRNA expression profiles with tissues of origin of the NCI-60 cell-lines, however, has been noted to be somewhat weaker than the association of mRNA profiles [22], [24]. With human tumor tissues, both mRNA and microRNA expression profiles appear to be similarly informative of tissue of origin [46], [47].

To compare the similarity of results obtained using different datasets, we also examined the association with population doubling-times of the NCI-60 cell-lines with their microRNA expression as measured by us or by Liu, et al [24]. Of the 28 microRNAs of our dataset that were identified as associated with doubling-time and were also quantified in the Liu study, 21 (75%) continued to be associated, none with a reversal of expression trend, when measurements were taken from the Liu dataset (table S4). Such similarity between the multiple NCI-60 microRNA datasets was also observed in correlation analysis of microRNA levels and radiation sensitivity of the cell-lines, in which microRNAs *let-7i*, *miR-142-5p* and *miR-193b* were identified as associated with at least one of the SF2, SF5 and SF8 radiation sensitivity parameters in datasets of this (table S3) as well as the Liu and Sokilde studies [23], [24]. Expression of *let-7i* and *miR-193b* positively correlated with SF2, SF5 and SF8, indicating radiation resistant cell-lines had higher levels of the two microRNAs. Expression of these two microRNAs has been shown to be induced by ionizing radiation [48], [49], [50]. Though microRNA *miR-142-5p* levels from all three datasets were negatively correlated with radiation sensitivity, expression of the microRNA has been observed to increase in response to ionizing radiation [50], [51], [52]. All three microRNAs, *let-7i*, *miR-142-5p* and *miR-193b*, are known to have growth-suppressive effects [53], [54], [55]. Unlike for expression of mRNAs [37], [56], microRNA expression patterns strongly associated with radiation sensitivity of the NCI-60 panel could not be discerned in the current study (figure S4).

As might be expected, mutations in the NCI-60 cells in three of the five oncogenes that were examined were found to be associated with microRNA expression changes (figure 4 and table S5). For example, *TP53* mutant cell-lines have higher levels of *miR-34a* and miR-34a* than the wild-type lines. The TP53 protein activates transcription of the *MIR34A* gene that encodes for the two mature microRNAs [57], [58]. For the *BRAF* gene, presence of an oncogenic mutation was found to be associated with 40 microRNAs (table S5). In the NCI-60 panel, there are 11 BRAF mutant cell-lines, including all eight melanoma lines. Indeed, except for miR-29b-1* (*miR-29b-1–5p*) and *miR-768-3p*, all microRNAs differentially expressed between the mutant and wild-type lines were also differentially expressed between the eight melanoma and 52 non-melanoma cell-lines. Nevertheless, the observed associations may indicate a universal role of the BRAF pathway in regulation of certain microRNAs. This is supported by the observation that *miR-146a*, whose levels are higher in *BRAF* mutant cell-lines (table S5), is upregulated upon activation of the *BRAF*-encoded B-raf kinase protein in non-melanoma cells [59]. Though *miR-29b* has been shown to target *PTEN* transcripts [60], a basis for the differential expression of the two microRNAs, *miR-29b* and *miR-769-3p*, between the *PTEN* mutant and wild-type lines (figure 4) is not evident in published literature. This exemplifies the value of NCI-60 integromics to generate testable hypotheses. Another potential use of the NCI-60 microRNA expression datasets such as the one generated here is for the identification of microRNA targets and molecular pathways through integrated analysis of gene expression data. One such analysis, depicted in figure S5, shows the presence of many significantly correlated pairs of microRNAs and target mRNAs predicted by nucleotide sequence comparison. As has been observed by others [61], a large fraction of such pairs had positive correlation values, which is paradoxical considering the prevalent notion of microRNA-mediated gene regulation, that microRNAs either abate target mRNA levels or not but they do not increase them. Such contradiction, which has also been observed with the NCI-60 datasets with other analytical approaches for microRNA-mRNA interaction [62], could reflect co-expression of microRNA and mRNA genes or indirect gene regulation, imperfectness of the TargetScan microRNA target prediction algorithm [63], or the fact that an mRNA molecule can be targeted by multiple species of microRNAs.

## Supporting Information

Figure S1 **Technical replication of microarray hybridizations.** Scatter-plots for four pairs of replicate hybridizations for RNA from four cell-lines are shown with ordered × values of log_2_-transformed microarray signal values (*dots*) and inter-replicate Pearson correlation coefficients, *r* (*lines*) for 1779 human RNA-specific probes. A rolling window of width 99 along the × axis was used to calculate r at the mid-window abscissa. Horizontal gray lines represent *Y = 0.5* along the right *Y* axis. Names of the cell-lines and the batches of microarray hybridizations are noted.
(TIF)

Figure S2 **Correlations between different NCI-60 cell-line microRNA expression datasets.** Cumulative frequency distributions are shown for Pearson correlation coefficients (*r*) with a bin-size of 0.025 for microRNAs quantified in the study of Sokilde, et al. [23] (*left*) or that of Liu, et al. [24] (*right*) and in other similar studies including this one (*Patnaik*) and those of Blower, et al. [22] and Gaur, et al [21]. The distributions of the coefficients with the expression measurements of Liu, et al. resampled are also shown. Numbers within parentheses in the legends indicate sample sizes, i.e., the number of microRNAs quantified in both of the compared datasets.
(TIF)

Figure S3 **Gaussian kernel density estimates of seven radiation survival parameter values of 59 NCI-60 cell-lines.** Data on the parameters SF2, SF5, SF8, D0, n, Casp8 and Casp16 were obtained from the study of Amundson, et al. [37]. Parameter values were log_2_-transformed and then z-score-normalized. The ks package (version 1.8.3) for R was used for density estimation with the package’s default settings. The short gray ticks along the × axis indicate parameter values of the 59 cell-lines.
(TIF)

Figure S4 **Distribution of Pearson coefficients in correlation analyses of microRNA expression and radiation sensitivity of 59 NCI-60 cell-lines.** Data on the radiation sensitivity parameters SF2, SF5, SF8, D0, n, Casp8 and Casp16 were obtained from the study of Amundson, et al. [37]. Expression measurements of 365, 495 and 896 microRNAs were respectively from the study of Liu, et al. [24], current study (*Patnaik*), and the study of Sokilde, et al. [23] as indicated in the figure. Leukemia cell-lines (n = 6) were excluded from the analyses depicted in the bottom half of the figure. All values were log_2_-transformed before correlation analyses. Pearson coefficients (*r*) are binned with a width of 0.02. Curve smoothing was done using four neighboring values and a zero-order polynomial.
(TIF)

Figure S5 **Distribution of Pearson coefficients for significant correlations of expression levels of microRNAs and their target mRNAs in 57 NCI-60 cell-lines.** Pearson coefficients (*r*) are binned with a width of 0.02. Curve smoothing was done using four neighboring values and a zero-order polynomial.
(TIF)

Figure S6 **Heat-map, with its pseudo-color scale underneath, of log_2_-transformed microarray signal values of the 60 cell-lines for the sets of eight microRNAs each with lowest P values in tests of differential expression in cell-lines of a specific tissue of origin compared to all the other cell-lines.** Both cell-lines and microRNAs are grouped by tissue of origin.
(TIF)

Table S1 **The 60 NCI-60 cell-lines examined in this work listed along with the day and batch of microarray hybridizations.**
(PDF)

Table S2 **Differential expression of microRNAs in NCI-60 cell-lines by tissue of origin.**
(PDF)

Table S3 **MicroRNAs whose expression correlates with an absolute Pearson correlation coefficient >0.4 for at least one of the radiation sensitivity parameters SF2, SF5 or SF8 in 53 non-leukemia NCI-60 cell-lines.**
(PDF)

Table S4 **MicroRNAs whose expression correlates with doubling-time of 59 NCI-60 cell-lines.**
(PDF)

Table S5 **MicroRNAs differentially expressed between NCI-60 cell-lines with and without BRAF gene mutations.**
(PDF)
