# Supplementary figures and images for: Expression of MicroRNAs in the NCI-60 Cancer Cell-Lines
Source: PLoS One. 2012 Nov 28;7(11):e49918. doi: 10.1371/journal.pone.0049918 (PMC3509128; doi:10.1371/journal.pone.0049918)

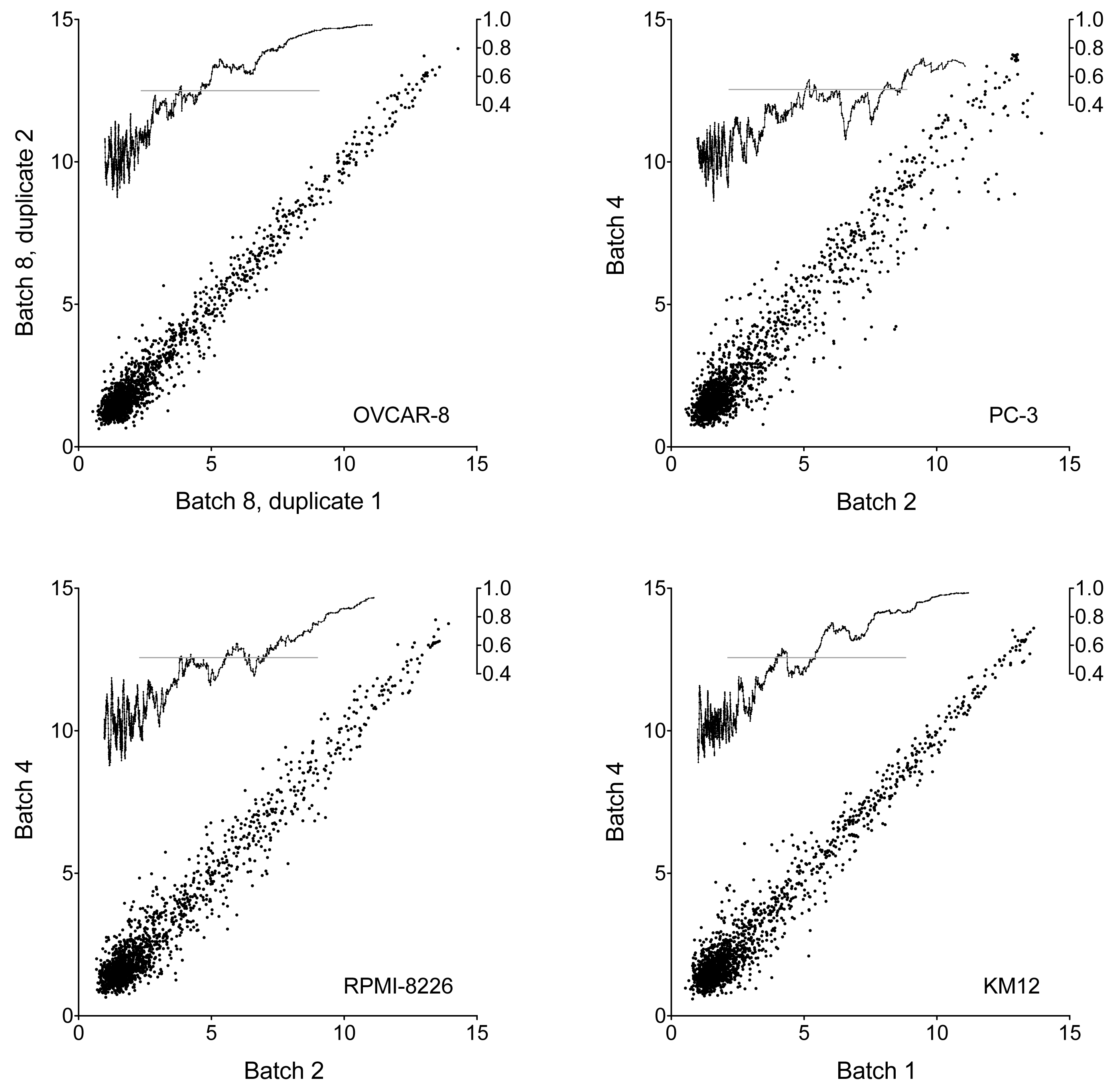

Supplement: Figure S1 — Technical replication of microarray hybridizations. Scatter-plots for four pairs of replicate hybridizations for RNA from four cell-lines are shown with ordered × values of log2-transformed microarray signal values (dots) and inter-replicate Pearson correlation coefficients, r (lines) for 1779 human RNA-specific probes. A rolling window of width 99 along the × axis was used to calculate r at the mid-window abscissa. Horizontal gray lines represent Y = 0.5 along the right Y axis. Names of the cell-lines and the batches of microarray hybridizations are noted. (TIF) [file pone.0049918.s001.tif]

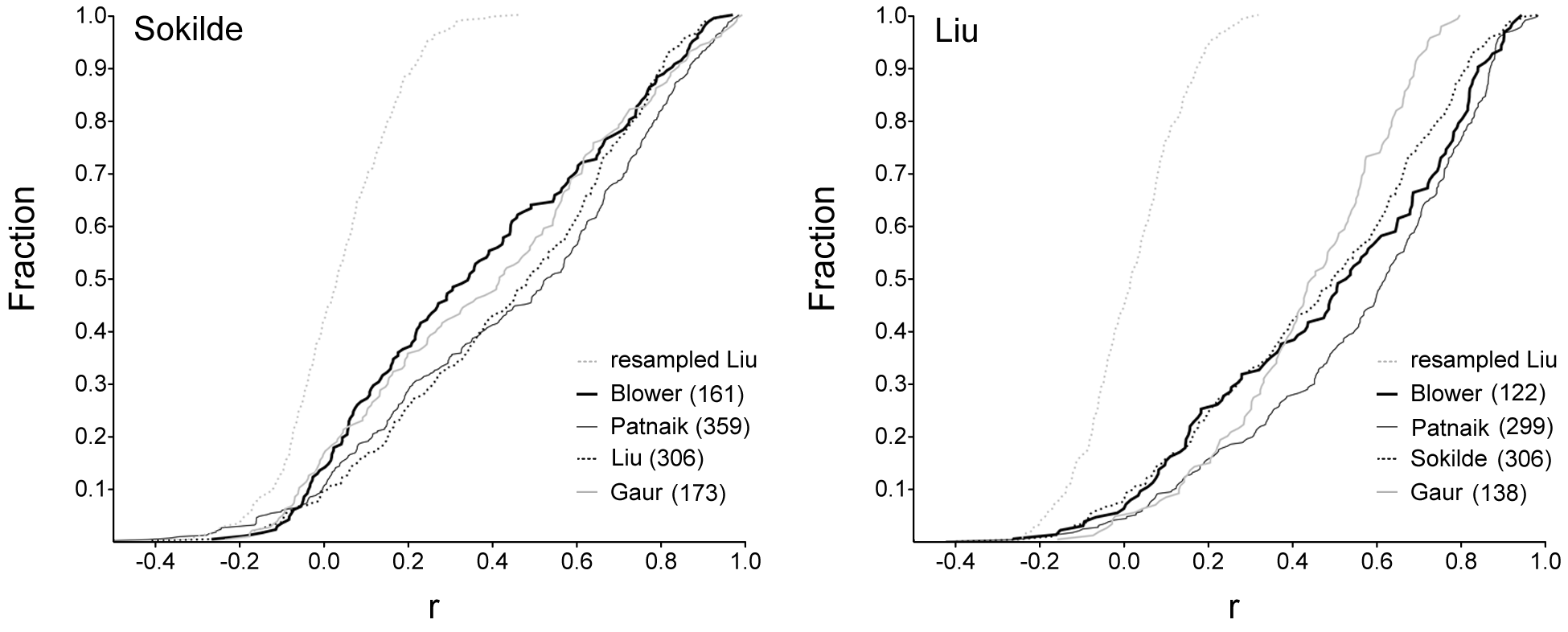

Supplement: Figure S2 — Correlations between different NCI-60 cell-line microRNA expression datasets. Cumulative frequency distributions are shown for Pearson correlation coefficients (r) with a bin-size of 0.025 for microRNAs quantified in the study of Sokilde, et al. [23] (left) or that of Liu, et al. [24] (right) and in other similar studies including this one (Patnaik) and those of Blower, et al. [22] and Gaur, et al [21]. The distributions of the coefficients with the expression measurements of Liu, et al. resampled are also shown. Numbers within parentheses in the legends indicate sample sizes, i.e., the number of microRNAs quantified in both of the compared datasets. (TIF) [file pone.0049918.s002.tif]

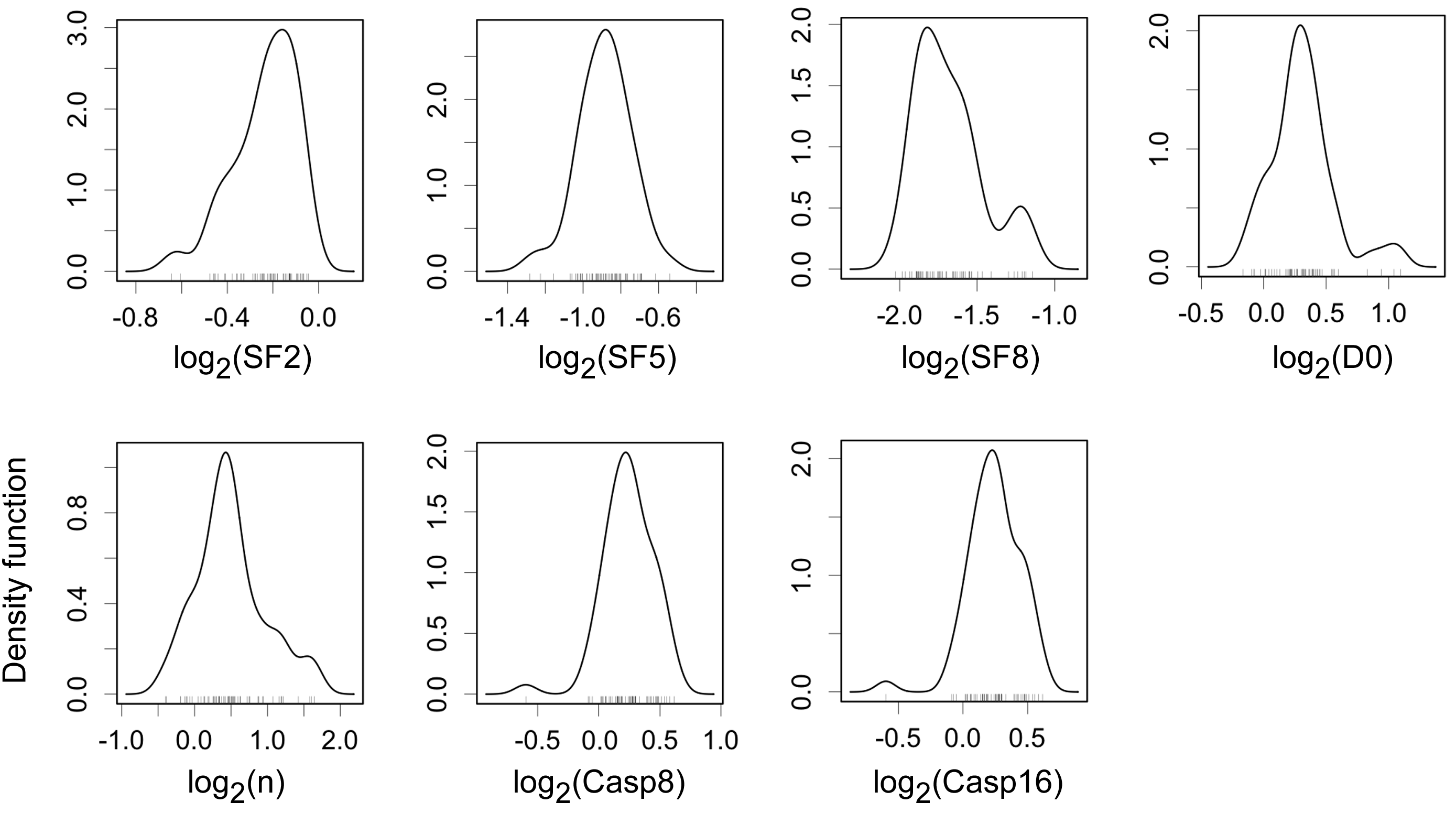

Supplement: Figure S3 — Gaussian kernel density estimates of seven radiation survival parameter values of 59 NCI-60 cell-lines. Data on the parameters SF2, SF5, SF8, D0, n, Casp8 and Casp16 were obtained from the study of Amundson, et al. [37]. Parameter values were log2-transformed and then z-score-normalized. The ks package (version 1.8.3) for R was used for density estimation with the package’s default settings. The short gray ticks along the × axis indicate parameter values of the 59 cell-lines. (TIF) [file pone.0049918.s003.tif]

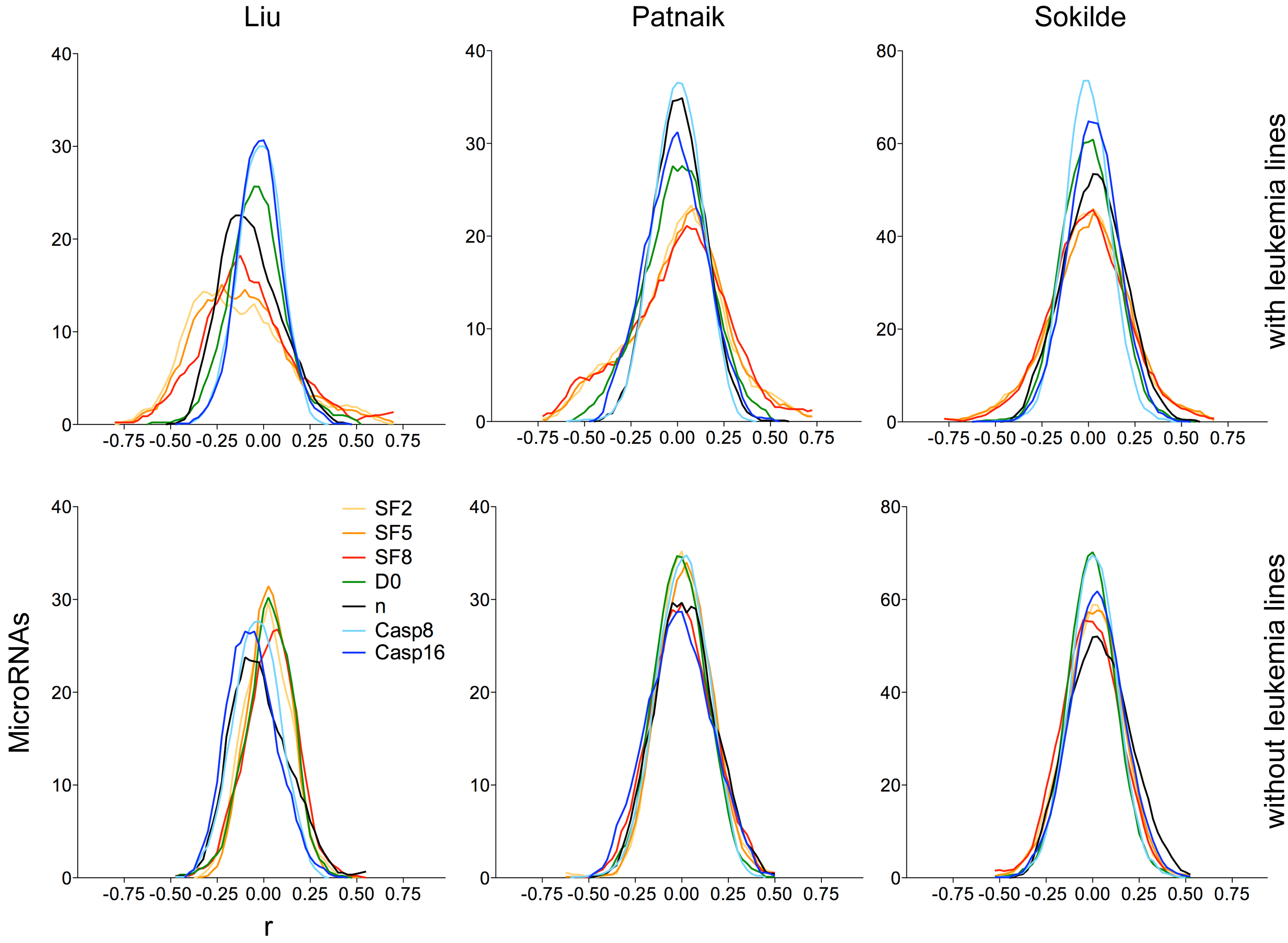

Supplement: Figure S4 — Distribution of Pearson coefficients in correlation analyses of microRNA expression and radiation sensitivity of 59 NCI-60 cell-lines. Data on the radiation sensitivity parameters SF2, SF5, SF8, D0, n, Casp8 and Casp16 were obtained from the study of Amundson, et al. [37]. Expression measurements of 365, 495 and 896 microRNAs were respectively from the study of Liu, et al. [24], current study (Patnaik), and the study of Sokilde, et al. [23] as indicated in the figure. Leukemia cell-lines (n = 6) were excluded from the analyses depicted in the bottom half of the figure. All values were log2-transformed before correlation analyses. Pearson coefficients (r) are binned with a width of 0.02. Curve smoothing was done using four neighboring values and a zero-order polynomial. (TIF) [file pone.0049918.s004.tif]

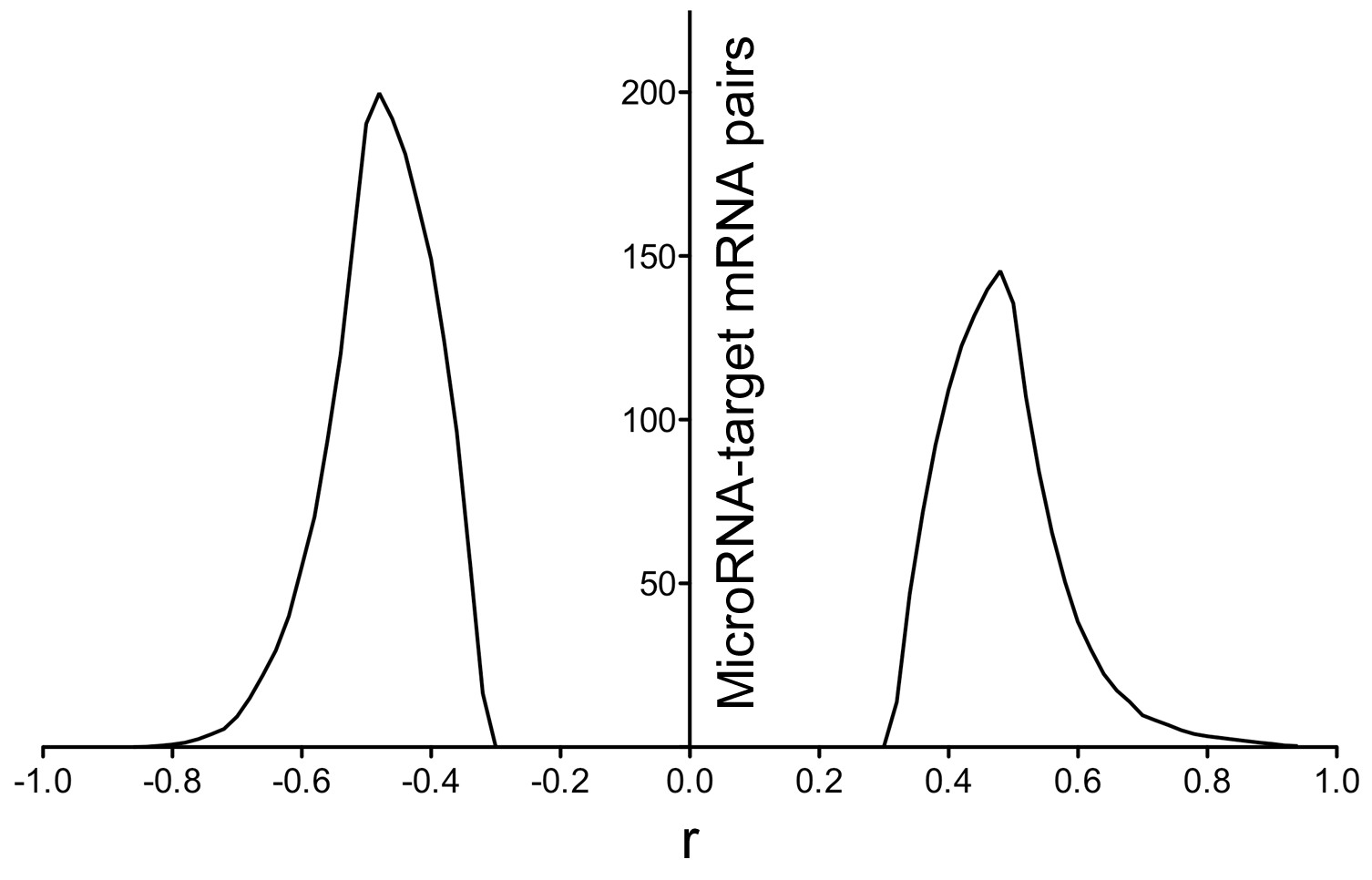

Supplement: Figure S5 — Distribution of Pearson coefficients for significant correlations of expression levels of microRNAs and their target mRNAs in 57 NCI-60 cell-lines. Pearson coefficients (r) are binned with a width of 0.02. Curve smoothing was done using four neighboring values and a zero-order polynomial. (TIF) [file pone.0049918.s005.tif]

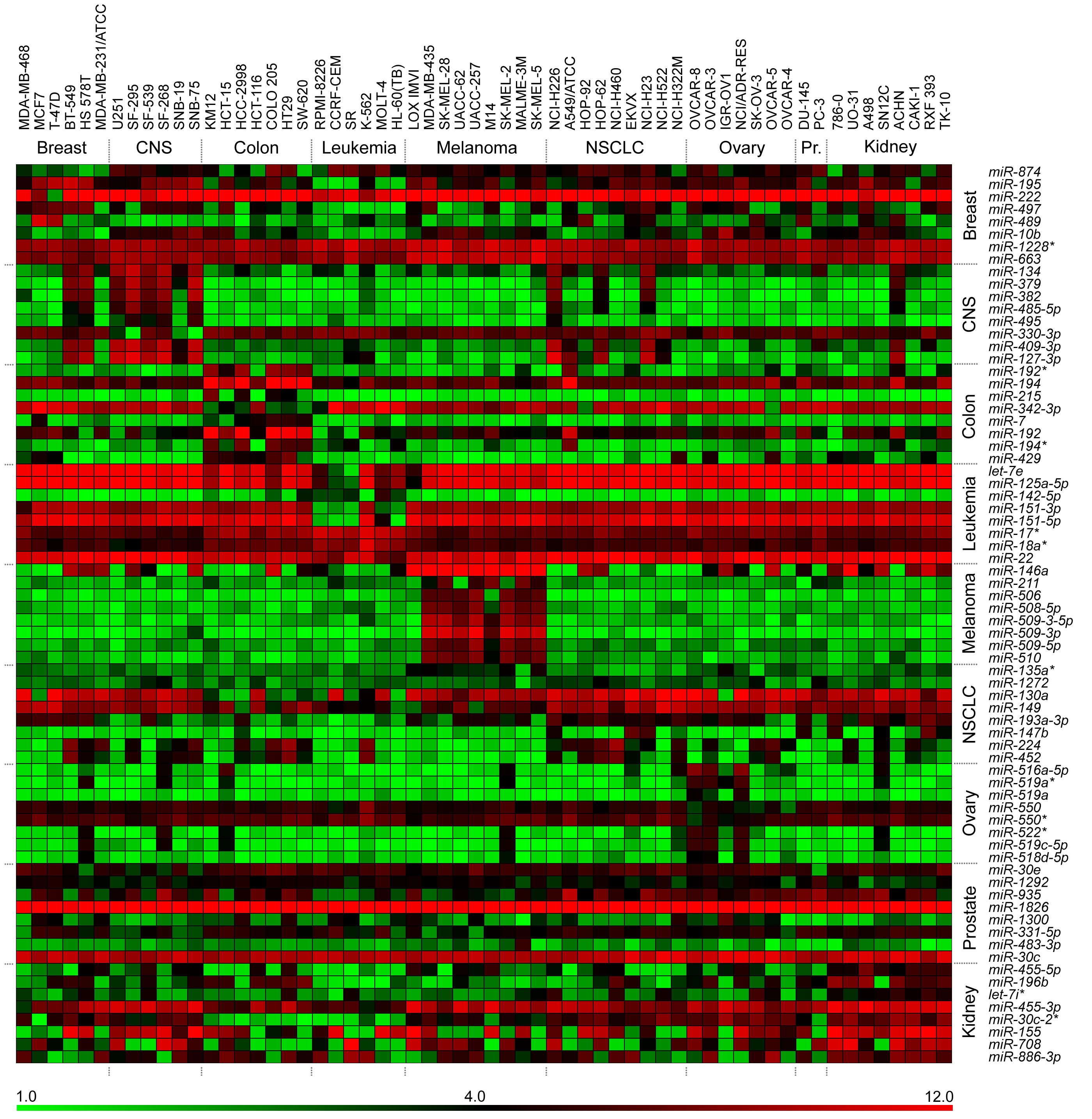

Supplement: Figure S6 — Heat-map, with its pseudo-color scale underneath, of log2-transformed microarray signal values of the 60 cell-lines for the sets of eight microRNAs each with lowest P values in tests of differential expression in cell-lines of a specific tissue of origin compared to all the other cell-lines. Both cell-lines and microRNAs are grouped by tissue of origin. (TIF) [file pone.0049918.s006.tif]
