# Supplementary material for: Expression of MicroRNAs in the NCI-60 Cancer Cell-Lines
Source: PLoS One. 2012 Nov 28;7(11):e49918. doi: 10.1371/journal.pone.0049918 (PMC3509128; doi:10.1371/journal.pone.0049918)
Supplement: Table S1 — The 60 NCI-60 cell-lines examined in this work listed along with the day and batch of microarray hybridizations. (PDF) [file pone.0049918.s007.pdf]

Table S1. The 60 NCI-60 cell-lines examined in this work listed along with the day and batch of microarray hybridizations[a]

Name	*Cancer tissue of origin*	*Cancer histology*	*Batch/day of microarray hybridization(s)*
KM12	Colon	Adenocarcinoma	1/1, 4/2, 8/3, 9/3
A549/ATCC	Lung	Adenocarcinoma	1/1
MDA-MB-468	Breast	Carcinoma	1/1
NCI-H322M	Lung	Adenocarcinoma	1/1
PC-3	Prostate	Carcinoma	2/1, 3/2, 6/2, 9/3
CAKI-1	Kidney	Adenocarcinoma	1/1
OVCAR-4	Ovary	Adenocarcinoma	1/1
RXF 393	Kidney	Hypernephroma	1/1
TK-10	Kidney	Carcinoma	1/1
RPMI-8226	Leukemia	Multiple myeloma	2/1, 3/2, 7/3, 9/3
U251	Central nervous system	Glioblastoma	2/1
CCRF-CEM	Leukemia	Acute lymphoblastic leukemia	2/1
SR	Leukemia	Immunoblastic large cell lymphoma	2/1
HCT-15	Colon	Adenocarcinoma	2/1
HCC-2998	Colon	Adenocarcinoma	2/1
LOX IMVI	Melanoma	Malignant amelanotic melanoma	2/1
OVCAR-8	Ovary	Adenocarcinoma	5/2, 8/3, 8/3, 9/3
786-0	Kidney	Adenocarcinoma	3/2
NCI-H23	Lung	Squamous cell carcinoma	3/2
HOP-92	Lung	Undifferentiated large cell carcinoma	3/2
HOP-62	Lung	Adenocarcinoma	3/2
UO-31	Kidney	Carcinoma	3/2
DU-145	Prostate	Carcinoma	3/2
OVCAR-3	Ovary	Ovary carcinoma	4/2

SF-295	Central nervous system	Glioblastoma multiforme	4/2
K-562	Leukemia	Chronic mylogenous leukemia	4/2
SF-539	Central nervous system	Gliosarcoma	4/2
MOLT-4	Leukemia	Acute lymphoblastic leukemia	4/2
SF-268	Central nervous system	Anaplastic astrocytoma	4/2
IGR-OV1	Ovary	Cystoadenocarcinoma	4/2
MDA-MB-435	Melanoma	Melanoma	5/2
MCF7	Breast	Adenocarcinoma	5/2
NCI-H522	Lung	Large cell carcinoma	5/2
HCT-116	Colon	Carcinoma	5/2
EKVX	Lung	Adenocarcinoma	5/2
NCI-H226	Ovary	Adenocarcinoma	5/2
A498	Kidney	Carcinoma	5/2
T-47D	Breast	Infiltrating ductal carcinoma	6/2
SK-MEL-28	Melanoma	Malignant melanoma	6/2
SN12C	Kidney	Carcinoma	6/2
BT-549	Breast	Infiltrating ductal carcinoma	6/2
UACC-62	Melanoma	Malignant melanoma	6/2
UACC-257	Melanoma	Malignant melanoma	6/2
COLO 205	Colon	Adenocarcinoma	6/2
HS 578T	Breast	Ductal carcinoma	7/3
SK-OV-3	Ovary	Adenocarcinoma	7/3
ACHN	Kidney	Carcinoma	7/3
NCI/ADR-RES	Ovary	Adenocarcinoma	7/3
M14	Melanoma	Amelanotic melanoma	7/3
HL-60(TB)	Leukemia	Promyelocytic leukemia	7/3
MDA-MB-231/ATCC	Breast	Adenocarcinoma	7/3
HT29	Colon	Adenocarcinoma	8/3
SW-620	Colon	Adenocarcinoma	8/3
SK-MEL-2	Melanoma	Malignant melanoma	8/3
MALME-3M	Melanoma	Malignant melanoma	8/3
SK-MEL-5	Melanoma	Malignant melanoma	8/3

SNB-19	Central nervous system	Glioblastoma	9/3
NCI-H460	Lung	Bronchioloalveolar carcinoma	9/3
OVCAR-5	Ovary	Carcinoma	9/3
SNB-75	Central nervous system	Astocytoma	9/3

[a]Names of cell-lines, cancer tissues of origin, and histology of the cancer tissues were obtained using the CellMiner™ web application of National Cancer Institute, USA.
