# Supplementary material for: Expression of MicroRNAs in the NCI-60 Cancer Cell-Lines
Source: PLoS One. 2012 Nov 28;7(11):e49918. doi: 10.1371/journal.pone.0049918 (PMC3509128; doi:10.1371/journal.pone.0049918)
Supplement: Table S2 — Differential expression of microRNAs in NCI-60 cell-lines by tissue of origin. (PDF) [file pone.0049918.s008.pdf]

Table S2. Differential expression of microRNAs in NCI-60 cell-lines by tissue of origin[a]

	Mean (range; standard deviation)		*Fold-change*	*t*	*P*	*Adj. P*
	Breast (n = 6)	Other (n = 54)				
miR-874	2.7 (1.7-3.6; 0.7)	5 (1.7-8; 1.6)	0.2	-3.59	0.0007	0.3279
miR-195	8 (4.8-10.5; 2.5)	5.3 (1.2-9.5; 2.1)	9.8	2.99	0.0040	0.5467
miR-222	10.3 (2.2-13.6; 4.4)	12.2 (8.7-13.5; 0.9)	0.9	-2.97	0.0042	0.5467
miR-497	6 (1.7-7.8; 2.3)	3.7 (1.1-7; 1.8)	8.6	2.96	0.0044	0.5467
miR-489	5 (2-10.9; 4)	2.8 (1-7.4; 1.5)	1.4	2.87	0.0056	0.5570
miR-10b	2.1 (1.1-3.2; 0.8)	4.2 (1.4-9.3; 1.9)	0.2	-2.79	0.0070	0.5763
*miR-1228**	8.4 (7.5-8.9; 0.6)	9.4 (7.9-11.2; 0.8)	0.7	-2.66	0.0100	0.6177
miR-663	7.8 (6.7-8.8; 0.7)	9 (6.8-11.4; 1)	0.5	-2.66	0.0099	0.6177
	CNS (n = 6)	Other (n = 54)				
miR-134	8.4 (4.9-10.3; 1.9)	3.4 (1.3-8.4; 1.7)	55.7	6.86	<0.0001	<0.0001
miR-379	7.9 (3.8-10; 2.3)	2.1 (0.9-8.4; 1.9)	119.4	7	<0.0001	<0.0001
miR-382	7.2 (2.3-9.7; 2.7)	1.9 (0.6-7.6; 1.7)	84.4	6.89	<0.0001	<0.0001
miR-485-5p	5.8 (1.9-8.3; 2.1)	1.9 (1-5.8; 1.1)	22.6	7.5	<0.0001	<0.0001
miR-495	4.2 (1.7-6; 1.7)	1.7 (0.9-4.4; 0.7)	8.0	7.05	<0.0001	<0.0001
miR-330-3p	3.6 (0.9-7.3; 2.7)	6.9 (4.6-8.5; 1)	0.1	-6.48	<0.0001	<0.0001
miR-409-3p	8.8 (4.7-10.3; 2.1)	3.3 (1.4-9.5; 1.9)	147.0	6.7	<0.0001	<0.0001
miR-127-3p	9.5 (5.1-11.3; 2.2)	2.9 (0.8-11.3; 2.6)	315.2	6.1	<0.0001	<0.0001
	Colon (n = 7)	Other (n = 54)				
*miR-192**	6.8 (1.6-8.8; 2.7)	2.2 (1-7.3; 1.1)	73.5	8.8	<0.0001	<0.0001
miR-194	12 (7.4-13.9; 2.4)	7 (4.1-12.3; 1.5)	104.0	7.93	<0.0001	<0.0001
miR-215	3.7 (0.9-7.7; 2.4)	1.3 (0.7-2; 0.3)	7.0	7.01	<0.0001	<0.0001
miR-342-3p	3.9 (2.3-8.5; 2.1)	9.3 (2.2-13; 2)	0.0	-6.97	<0.0001	<0.0001
miR-7	4.3 (2-5.8; 1.4)	1.7 (1-4.7; 0.6)	8.0	8.9	<0.0001	<0.0001
miR-192	10.4 (4.3-12.7; 3.1)	5.1 (1.3-10.6; 2)	111.4	6.43	<0.0001	<0.0001
*miR-194**	5.5 (1-7.6; 2.5)	2.1 (0.9-7.6; 1.1)	24.3	6.49	<0.0001	<0.0001
miR-429	5.3 (2.8-7.6; 1.6)	2 (0.7-5.8; 1.3)	14.9	6.24	<0.0001	<0.0001
	Leukemia (n = 6)	Other (n = 54)				
let-7e	6.2 (1.3-11.1; 3.6)	11.6 (5.5-13; 1.3)	0.0	-8.06	<0.0001	<0.0001
miR-125a-5p	6.4 (2.7-11.6; 3.4)	11.5 (4-12.9; 1.4)	0.0	-7.25	<0.0001	<0.0001
miR-142-5p	3.3 (1.1-5.6; 1.4)	1.4 (0.5-3.7; 0.5)	3.7	6.74	<0.0001	<0.0001
miR-151-3p	3.3 (1.7-9; 2.8)	9 (5.6-10.6; 0.8)	0.0	-11.57	<0.0001	<0.0001
miR-151-5p	3.7 (1.2-11.3; 4)	11.1 (9.2-12.3; 0.6)	0.0	-13.5	<0.0001	<0.0001
*miR-17**	10.2 (9.2-11; 0.7)	7.3 (5.2-9.7; 1)	8.0	6.79	<0.0001	<0.0001
*miR-18a**	8.8 (7.6-11.1; 1.3)	6 (3.7-7.5; 0.8)	5.7	7.32	<0.0001	<0.0001
miR-22	8.5 (6.4-10.7; 1.8)	11.3 (9.7-13.4; 0.9)	0.1	-6.58	<0.0001	<0.0001
	Melanoma (n = 9)	Other (n = 54)				
miR-146a	12.6 (12.1-13; 0.3)	4.2 (0.9-12; 3.5)	1024.0	7.35	<0.0001	<0.0001
miR-211	4.8 (1.5-7.8; 2.5)	2.2 (1.1-3.7; 0.5)	9.8	6.87	<0.0001	<0.0001
miR-506	5.8 (1.9-8.3; 2.2)	1.4 (0.8-2.2; 0.3)	36.8	14.03	<0.0001	<0.0001

miR-508-5p	6.3 (1.6-8.3; 2.3)	1.9 (1-2.9; 0.4)	45.3	12.81	<0.0001	<0.0001
miR-509-3-5p	7.5 (1.1-9.7; 2.8)	1.7 (0.8-3.3; 0.5)	90.5	14.35	<0.0001	<0.0001
miR-509-3p	9.2 (1-11.7; 3.4)	1.3 (0.7-3.3; 0.4)	548.7	16.72	<0.0001	<0.0001
miR-509-5p	6.4 (2.3-8.8; 2.1)	2.2 (1.1-3.2; 0.5)	22.6	12.95	<0.0001	<0.0001
miR-510	6.4 (1.9-8; 2.1)	1.9 (1-3.3; 0.5)	45.3	13.82	<0.0001	<0.0001
	NSCLC (n = 9)	Other (n = 54)				
*miR-135a**	2.1 (1.6-2.7; 0.4)	2.8 (1.4-4.7; 0.7)	0.6	-3	0.0039	0.8223
miR-1272	3.3 (2.3-4.1; 0.5)	2.7 (1.4-4.3; 0.6)	1.5	2.48	0.0158	0.8223
miR-130a	10.8 (9.3-12.2; 1)	8.1 (1.6-12.2; 3.4)	2.3	2.47	0.0161	0.8223
miR-149	8.9 (6-11.1; 1.4)	7.2 (2.5-10.6; 2.1)	2.3	2.43	0.0181	0.8223
miR-193a-3p	5.4 (4.3-6.7; 0.8)	3.9 (0.9-7.6; 1.9)	4.0	2.4	0.0193	0.8223
miR-147b	3 (1.1-7.1; 2.3)	1.8 (0.7-7.2; 1.3)	1.2	2.38	0.0202	0.8223
miR-224	4.9 (1.1-9; 2.3)	3.1 (0.8-8.7; 2.2)	12.1	2.35	0.0220	0.8223
miR-452	4.3 (1.5-7.4; 2.1)	2.7 (0.9-7.8; 1.9)	10.6	2.35	0.0220	0.8223
	Ovary (n = 7)	Other (n = 54)				
miR-516a-5p	4.8 (1.5-8.2; 2.8)	1.7 (0.7-6.3; 1.1)	4.3	5.46	<0.0001	0.0003
*miR-519a**	3.9 (1.5-6.2; 2.1)	1.5 (0.6-4.9; 1)	6.5	5.35	<0.0001	0.0003
miR-519a	2.6 (1.1-5.2; 1.7)	1.4 (0.5-2.5; 0.4)	1.2	4.68	<0.0001	0.0020
miR-550	3.9 (2.6-5.1; 0.9)	5.4 (3.6-8.2; 0.8)	0.3	-4.67	<0.0001	0.0020
*miR-550**	4.9 (3.3-6; 1)	6.4 (4.4-8.5; 0.8)	0.4	-4.61	<0.0001	0.0020
*miR-522**	3.6 (1.1-6; 2.2)	1.6 (0.7-5.3; 1)	2.3	4.14	0.0001	0.0090
miR-519c-5p	3.6 (1.4-6; 2.2)	1.6 (0.7-5.1; 1)	1.7	4.08	0.0001	0.0094
miR-518d-5p	2.9 (1.1-5.2; 1.7)	1.6 (0.9-4.2; 0.6)	2.5	3.95	0.0002	0.0120
	Prostate (n = 2)	Other (n = 54)				
miR-30e	3.7 (1.9-5.4; 2.5)	5.8 (2.6-7.7; 1.1)	0.2	-2.68	0.0095	0.9950
miR-1292	5.2 (4.3-6.1; 1.3)	4.1 (2.2-5.5; 0.7)	2.0	2.07	0.0423	0.9950
miR-935	8.1 (7.5-8.7; 0.8)	5.3 (2.3-10; 2)	6.5	2.05	0.0447	0.9950
miR-1826	11.8 (11.7-11.9; 0.1)	12.4 (11.6-13.3; 0.4)	0.7	-2.03	0.0468	0.9950
miR-1300	1.4 (1.2-1.6; 0.3)	2.9 (1-7; 1.2)	0.5	-1.78	0.0794	0.9950
miR-331-5p	6 (6-6.1; 0.1)	4.6 (2.2-6.6; 1.1)	2.3	1.78	0.0799	0.9950
miR-483-3p	1.4 (1.3-1.4; 0.1)	2.2 (0.9-5.7; 0.7)	0.5	-1.74	0.0870	0.9950
miR-30c	8.9 (8.7-9.1; 0.2)	9.9 (8.5-11.6; 0.9)	0.5	-1.72	0.0905	0.9950
	Kidney (n = 8)	Other (n = 54)				
miR-455-5p	5.5 (4.1-7.7; 1.1)	2.8 (1.1-5.6; 1.4)	8.6	5.22	<0.0001	0.0011
miR-196b	6.6 (1.3-8.6; 2.3)	3.4 (0.9-7.6; 1.9)	21.1	4.43	<0.0001	0.0096
*let-7i**	4.7 (3.5-5.9; 0.8)	2.9 (1.3-6.1; 1.1)	4.0	4.27	0.0001	0.0114
miR-455-3p	11.1 (10.4-12.7; 0.8)	8.5 (3.4-11.4; 2)	4.0	3.71	0.0005	0.0560
*miR-30c-2**	7.4 (5.6-8.8; 1.2)	4.6 (0.9-8.9; 2.3)	7.0	3.51	0.0009	0.0844
miR-155	10.8 (8.3-12.8; 1.4)	6.2 (1.4-12.5; 3.9)	13.9	3.39	0.0012	0.1005
miR-708	8.4 (1.5-11.6; 3.5)	4.6 (1.2-12.2; 3.1)	59.7	3.25	0.0019	0.1322
miR-886-3p	7.1 (4.8-8.2; 1.1)	4.6 (1.4-9; 2.2)	6.5	3.14	0.0026	0.1535

[a]Differential expression was evaluated with empirical Bayes moderated t statistics provided with the limma Bioconductor package (version 3.10.0) in R. Log_2-transformed microarray signal values were used. The eight microRNAs with the lowest P values in differential expression analyses in cell-lines of a particular tissue of origin compared to the rest of the 60 NCI-60 cell-lines are tabulated along with fold-change values (ratio of intra-group medians of untransformed microarray signal values), t statistics, unadjusted P values, and P values

adjusted by the Benjamini-Hochberg method for a maximum false discovery rate of 5% in inter-group comparisons. Intra-group means, ranges and standard deviations of log_2-transformed microarray signal values are also shown.
