# Supplementary material for: Expression of MicroRNAs in the NCI-60 Cancer Cell-Lines
Source: PLoS One. 2012 Nov 28;7(11):e49918. doi: 10.1371/journal.pone.0049918 (PMC3509128; doi:10.1371/journal.pone.0049918)
Supplement: Table S3 — MicroRNAs whose expression correlates with an absolute Pearson correlation coefficient >0.4 for at least one of the radiation sensitivity parameters SF2, SF5 or SF8 in 53 non-leukemia NCI-60 cell-lines. (PDF) [file pone.0049918.s009.pdf]

**Table S3.** MicroRNAs whose expression correlates with an absolute Pearson correlation coefficient >0.4 for at least one of the radiation sensitivity parameters SF2, SF5 or SF8 in 53 non-leukemia NCI-60 cell-lines[a]

| | *Pearson r* | | |
|---|---|---|---|
| | *SF2* | *SF5* | *SF8* |
| Liu | | | |
| *let-7i* | 0.42 | 0.37 | 0.40 |
| *miR-142-3p* | -0.04 | -0.16 | -0.45 |
| *miR-142-5p* | 0.00 | -0.12 | -0.43 |
| *miR-193b* | 0.28 | 0.31 | 0.42 |
| Patnaik | | | |
| *let-7e* | 0.45 | 0.35 | 0.33 |
| *let-7i* | 0.43 | 0.40 | 0.41 |
| *let-7i** | 0.32 | 0.38 | 0.46 |
| *miR-125a-5p* | 0.43 | 0.33 | 0.31 |
| *miR-142-5p* | -0.33 | -0.38 | -0.41 |
| *miR-193b* | 0.36 | 0.37 | 0.44 |
| *miR-193b** | 0.33 | 0.40 | 0.45 |
| *miR-552* | -0.46 | -0.37 | -0.12 |
| *miR-592* | -0.62 | -0.57 | -0.39 |
| *miR-99b* | 0.40 | 0.33 | 0.30 |
| Sokilde | | | |
| *let-7g** | 0.43 | 0.41 | 0.23 |
| *let-7i* | 0.43 | 0.42 | 0.47 |
| *miR-103-2** | -0.46 | -0.39 | -0.26 |
| *miR-142-3p* | -0.46 | -0.45 | -0.50 |
| *miR-142-5p* | -0.45 | -0.46 | -0.51 |
| *miR-153* | -0.23 | -0.28 | -0.47 |
| *miR-193b* | 0.38 | 0.41 | 0.46 |
| *miR-217* | -0.18 | -0.26 | -0.42 |
| *miR-223* | -0.30 | -0.30 | -0.40 |
| *miR-488** | 0.48 | 0.48 | 0.31 |
| *miR-592* | -0.44 | -0.34 | -0.27 |
| *miR-625* | -0.34 | -0.43 | -0.51 |
| *miR-647* | 0.42 | 0.38 | 0.11 |
| *miRPlus-A1044* | -0.33 | -0.39 | -0.47 |

[a]$Log_2$-transformed data on the radiation sensitivity parameters were obtained from the study of Amundson, et al. (*Cancer Res*, 68:415-424). $Log_2$-transformed expression measurements of 365, 495 and 896 microRNAs were respectively from the studies of Liu, et al. (*Mol Cancer*

*Ther*, 9:1080-1091), Patnaik, et al. (current study), and Sokilde, et al. (*Mol Cancer Ther*, 10:375-384) as indicated in the table.
