# Supplementary material for: Expression of MicroRNAs in the NCI-60 Cancer Cell-Lines
Source: PLoS One. 2012 Nov 28;7(11):e49918. doi: 10.1371/journal.pone.0049918 (PMC3509128; doi:10.1371/journal.pone.0049918)
Supplement: Table S4 — MicroRNAs whose expression correlates with doubling-time of 59 NCI-60 cell-lines. (PDF) [file pone.0049918.s010.pdf]

Table S4. MicroRNAs whose expression correlates with doubling-time of 59 NCI-60 cell-lines [a]

	Patnaik		*Liu*	
	Score (d)	*q-value (%)*	*Score (d)*	*q-value (%)*
let-7e	2.14	0.00	1.65	9.21
miR-100	2.44	0.00	2.57	0.00
miR-125a-5p	2.01	0.00	2.41	0.00
miR-125b	3.02	0.00	3.12	0.00
*miR-125b-1**	2.08	0.00	NA	NA
miR-1270	1.69	11.26	NA	NA
miR-130a	2.66	0.00	3.16	0.00
miR-149	2.09	0.00	1.54	11.61
*miR-17**	-2.01	4.73	-2.58	0.00
miR-184	1.67	11.26	NA	NA
miR-18a	-1.83	11.26	-2.29	1.33
*miR-18a**	-2.11	4.73	NA	NA
*miR-192**	-2.04	4.73	-1.91	3.92
miR-193a-3p	2.71	0.00	2.30	0.00
miR-193a-5p	2.71	0.00	1.96	1.33
*miR-194**	-2.00	4.73	NA	NA
miR-19a	-2.25	4.73	-2.25	1.33
miR-19b	-1.91	8.75	-1.75	3.92
miR-22	1.66	11.26	2.38	0.00
*miR-24-2**	1.87	4.64		
miR-28-3p	1.76	8.75		
miR-28-5p	2.19	0.00	2.23	0.00
miR-297	-2.28	4.73		
miR-30a	3.51	0.00	4.03	0.00
*miR-30a**	3.76	0.00	3.26	0.00
*miR-30c-2**	3.45	0.00		
miR-34b	-2.17	4.73		
*miR-34b**	1.65	11.26	1.73	6.74
miR-34c-3p	2.09	0.00	NA	NA
miR-34c-5p	2.94	0.00		
miR-543	1.62	11.26		
miR-7	-1.97	4.73	-2.74	0.00
miR-99a	1.63	11.26	2.22	0.00
miR-99b	1.97	0.00	2.36	0.00
*miR-99b**	1.71	8.75	NA	NA

[a]The samr package (version 2.0) for R was used to perform significance analysis of microarrays (SAM; *Proc Natl Acad Sci U S A*, 98:5116-21) to identify microRNAs whose expression correlates with the doubling-time of 59 cell-lines of the NCI-60 panel. Doubling-time was treated as a continuous variable, log_2-transformed microarray signal values were

used, and estimated false discovery rate was limited to 10%. Otherwise, the default values for various parameters in samr were used. For the current study (Patnaik, et al.), 495 microRNAs were analyzed whereas the number was 365 for the study of Liu, et al. (*Mol Cancer Ther*, 9:1080-1091). Negative score (d) values indicate a negative association with doubling-time. Blank value for a microRNA indicates that it was not examined in the study of Liu, et al.; NA suggests that the microRNA was not identified as being associated with doubling-time in the SAM analysis.
