# Supplementary material for: Expression of MicroRNAs in the NCI-60 Cancer Cell-Lines
Source: PLoS One. 2012 Nov 28;7(11):e49918. doi: 10.1371/journal.pone.0049918 (PMC3509128; doi:10.1371/journal.pone.0049918)
Supplement: Table S5 — MicroRNAs differentially expressed between NCI-60 cell-lines with and without BRAF gene mutations. (PDF) [file pone.0049918.s011.pdf]

Table S5. MicroRNAs differentially expressed between NCI-60 cell-lines with and without *BRAF* gene mutations[a]

	Fold-change	*P*
miR-509-3p	5.31	<0.001
miR-509-3-5p	3.89	<0.001
miR-513a-5p	3.25	<0.001
miR-506	2.95	<0.001
miR-584	3.86	<0.001
miR-146a	6.68	<0.001
miR-509-5p	2.73	<0.001
miR-510	2.91	<0.001
miR-502-5p	1.73	<0.001
miR-508-5p	2.80	<0.001
miR-513c	2.09	<0.001
miR-211	1.99	0.0001
miR-513b	1.47	0.0002
miR-92b	-1.48	0.0003
miR-30d	1.03	0.0010
miR-508-3p	1.23	0.0022
*miR-425**	-1.20	0.0056
miR-501-5p	1.00	0.0056
*miR-30b**	1.12	0.0065
miR-660	1.58	0.0083
miR-30b	1.01	0.0084
miR-335	-0.99	0.0101
miR-185	1.08	0.0117
miR-149	-2.19	0.0171
miR-339-5p	-0.99	0.0171
miR-204	1.03	0.0216
miR-502-3p	1.38	0.0246
*miR-589**	-1.14	0.0246
*miR-500**	1.39	0.0294
miR-501-3p	1.37	0.0327
miR-768-5p	-0.72	0.0393
*miR-135a**	0.71	0.0433
*miR-181c**	-1.44	0.0433
miR-331-3p	-0.90	0.0433
miR-1296	-1.22	0.0434
miR-532-3p	1.20	0.0434
miR-532-5p	1.22	0.0434
miR-663	1.03	0.0434
miR-768-3p	-0.72	0.0434
*miR-29b-1**	1.54	0.0453

[a]Differential expression was evaluated with empirical Bayes moderated t statistics provided with the limma Bioconductor package (version 3.10.0) in R. Log_2-transformed microarray signal values were used. The 40 microRNAs that are the most differentially expressed ($P < 0.05$) in the group of 11 NCI-60 cell-lines with *BRAF* mutations compared to that of 48 without are tabulated along with fold-changes (difference of intra-group means) and P values adjusted by the Benjamini-Hochberg method for a maximum false discovery rate of 5%.
